# Long-Term Outcomes Stratified by Age in Patients with a Mechanical versus Biological Mitral Valve Replacement

**DOI:** 10.3390/jcdd9100339

**Published:** 2022-10-06

**Authors:** Gábor Veres, Kálmán Benke, Roland Stengl, Petra Weber, Ereva Marina, Gábor Szabó, Matthias Karck

**Affiliations:** 1 Department of Cardiac Surgery, Martin Luther University Halle-Wittenberg, Ernst-Grube Str. 40, 06120 Halle (Saale), Germany; 2 Heart and Vascular Center, Semmelweis University, Városmajor u. 68, 1122 Budapest, Hungary; 3 Department of Cardiac Surgery, University of Heidelberg, INF 326, 69120 Heidelberg, Germany

**Keywords:** cardiac surgery, heart valve prosthesis implantation, mitral valve insufficiency, mitral valve stenosis

## Abstract

Objectives: Balancing anticoagulation and reoperation risks determines prostheses choice (mechanical/biological) for mitral valve replacement. We aimed to re-evaluate the outcomes after biological versus mechanical mitral valve replacement. Methods: We compared long-term benefits and risks of mechanical and biological prostheses in 2056 patients (52% men, 48% women; 65.4 ± 12.1 years) who underwent mitral valve replacements between 1993–2017, in a retrospective single-centre study. Data sources included prospective institutional database, social registry, general practitioner data and follow-up questionnaire. Patients were stratified by age: < = 39 y (*n* = 82), 40–49 y (*n* = 164), 50–59 y (*n* = 335), 60–69 y (*n* = 593), 70–79 y (*n* = 743) and > = 80 y (*n* = 139). Long-term outcomes (mortality, reoperations, bleeding) were analysed. Results: Altogether, 1308 mechanical (53% men, 47% women; 61.5 ± 11.7 years) and 748 biological (50% men, 50% women; 72.3 ± 9.6 years) valves were implanted. The reason for valve replacement was stenosis in 162, insufficiency in 823 and combined in 323 cases for mechanical, while it was 46, 567 and 135 for biological valves, respectively. Overall cumulative survival was higher with mechanical prosthesis (mean: 139 ± 4 vs. 102 ± 5 months, 10 y: 55% vs. 33%, *p* < 0.0001). Subgroup analysis revealed higher survival among patients receiving mechanical prosthesis up to 60 years (< = 39 y *p* = 0.047, 40–49 y *p* < 0.0001, 50–59 y *p* = 0.001). In patients 60–69 years, overall survival did not differ; however, in survivors beyond 8 years, mechanical prosthesis showed improved survival (*p* = 0.014). While between 70–79 years survival was nearly identical, for above 80 years, patients had a higher survival with biological prosthesis (*p* = 0.014). Conclusion: The present data demonstrated a higher survival of mechanical prosthesis in a wide range of patients after mitral valve replacement.

## 1. Introduction

Valvular heart diseases are an important public health problem, mostly with a degenerative background in industrialised countries and mainly caused by rheumatic disease in developing countries [1]. According to the Euro Heart Survey, mitral regurgitation is one of the most common valvular heart diseases, followed by aortic regurgitation and mitral stenosis with an equivalent frequency. A majority of the patients are elderly with a high number of cardiovascular risk factors and comorbidities [2].

Based on the announcement from the German Society of Cardiothoracic and Vascular Surgery, the number of heart valve interventions increased in 2019 by around 5% to 36,650 compared to 2018, when this number was 34,915. In 2019, 6419 isolated mitral valve operations were performed; among them, 64.5% was mitral valve repair and 35.5% was implantation of biological or mechanical mitral valve [3].

Data on the choice of biological versus mechanical mitral valve are still controversial. Mechanical valves are thought to be associated with an increased risk of haemorrhagic/thromboembolic events because of life-long anticoagulation, but biological valves are associated with a higher risk of reoperation [4]. The choice between mechanical and biological valve in adults is determined mainly by estimating the risk of anticoagulation-related bleeding and thromboembolism with a mechanical valve versus the risk of structural valve deterioration with a bioprosthesis and by considering the patient’s lifestyle and preferences [5]. Biological prostheses have recently been increasingly favoured in patients aged 60 to 70 years, albeit with a low level of evidence.

Recently, mainly observational studies have shown equivalent mortality regardless of valve type or position, among patients 50 to 69 years of age [6,7]. These results support the increasing use of biological valves in younger patients, but aforementioned studies may have been underpowered to detect differences in long-term mortality. However, the choice between mechanical and biological valves for mitral valve replacement in patients under 70 years is less evident [6].

Therefore, the aim of the present study is to evaluate the long-term benefits and risks of mechanical and biological prostheses for mitral valve replacement in a single-centre retrospective cohort study involving patients undergoing mitral valve replacement at the University Hospital Heidelberg in the last 25 years.

## 2. Patients and Methods

### 2.1. Ethical Statement

Ethical review and approval were waived for this study due to the retrospective pseudonymised study design.

### 2.2. Study Design

In our single-centre retrospective study, we examined data from patients who underwent primary mitral valve replacement at the University of Heidelberg, Heidelberg, Germany between 1 January 1993 and 31 December 2017 to study the long-term effects of mechanical and biological prostheses. Data on survival, bleeding and reoperation were collected from the prospective institutional database, social registry and from general practitioner and cardiologist data. Furthermore, patients were contacted via phone or post, and questionnaires were applied to assess the quality of life, use of anticoagulants, the need for any interventions after the primary operation and any existing comorbidities of the patients during our routine institutional follow-up.

### 2.3. Study Population

Altogether, 2056 adult patients with isolated or combined (with another valve replacement or with coronary bypass surgery) mitral valve replacement were included. Mainly based on surgeon’s preference, minimal invasive or complete median sternotomy approach was carried out. The whole cohort was divided into two main groups according to the implanted valve type—biological (*n* = 748; Medtronic Mosaic, Carpentier-Edwards Magna Ease) or mechanical (*n* = 1308; bileaflet St. Jude medical prosthesis, Single tilting disc by Medtronic Mosaic, Caged-ball by Starr–Edwards). The median follow-up duration among recipients of biological prosthesis was 6.5 years (interquartile range, 5.9–7.1) and 15.1 years (interquartile range, 14.3–15.8) for recipients of mechanical prosthesis.

The patients were further stratified according to age as follows: under 39 years (*n* = 82), 40 to 49 years (*n* = 164), 50 to 59 years (*n* = 335), 60 to 69 years (*n* = 593), 70 to 79 (*n* = 743) years and above 80 years (*n* = 139). Moreover, the patients were characterised by the New York Heart Association (NYHA) Functional Classification. Patients with secondary mitral valve replacement (reoperation) and patients with secondary heart transplantation were excluded from the study.

### 2.4. Statistical Analysis

A retrospective analysis of prospectively collected data was performed using SPSS Statistics 24 (IBM Corp, Armonk, NY). Data are presented as percentages and number of cases. Chi-square test was applied to compare frequencies of comorbidities and taken medications of the two groups. The value of *p* < 0.05 was considered statistically significant. Continuously distributed variables were evaluated using Kaplan–Meier analyses. Additionally, descriptive statistic, Mantel–Cox, Breslow and Tarone–Ware analyses were performed by the statistical software.

## 3. Results

### 3.1. Preoperative and Intraoperative Characteristics

Of the included 2056 patients, 52% (*n* = 1070) were men and 48% (*n* = 986) were women. The average age at the time of the operation was 65.4 ± 12.1 years for the whole cohort, while it was 72.3 ± 9.6 years in the biological and 61.5 ± 11.7 years in the mechanical valve groups.

Table 1 demonstrates the occurrence of the consequences of the mitral valve disease, other comorbidities, the taken antithrombotic medications and NYHA functional classification before the operation.

**Figure 1:**
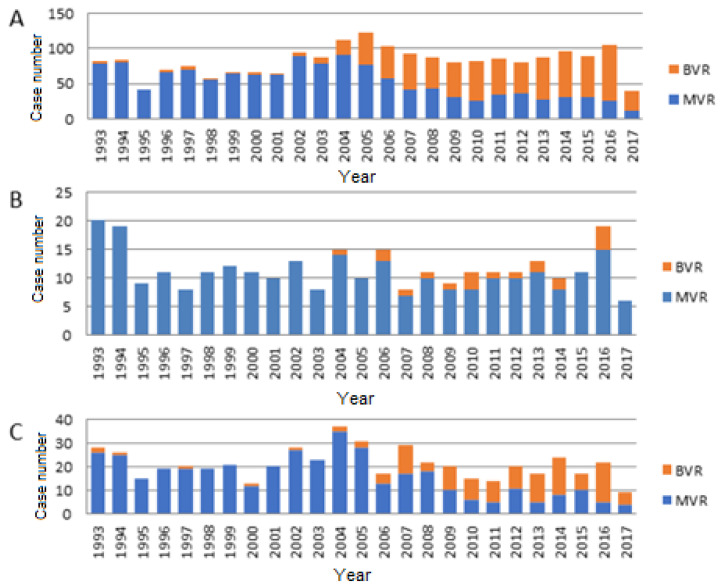
(**A**) The use of mechanical and biological valves in the whole cohort. Until 2003, the trend for the use of biological valves was consistently low. In 2004, its number slightly raised and after the change in guidelines the tendency is changed in favour of more than 50% usage of biological prosthesis per year. (**B**) Distribution of biological and mechanical valve implantations in patients between 50 and 59 years. Biological valves are used in a small percentage of patients undergoing mitral surgery in this age group. (**C**) Distribution of biological and mechanical valve implantations in patients between 60 and 69 years. The use of biological prosthesis started in the early 1990s, but there was about 10 years of non-favourisation. After the change in guidelines in 2006, more than half of the implanted valves were biological in this patient population. BVR = biological valve replacement; MVR = mechanical valve replacement.

The tendency of the use of mechanical and biological valves in our patients is demonstrated in Figure 1.

#### Survival

At the end of the 25-year period, 43.5% of the patients (*n* = 894) of the whole cohort were still alive. Of the 1162 deaths, 15.4% (*n* = 179) occurred due to cardiac cause, while the others were non-cardiac or unknown causes. From the group of patients receiving biological valves, 49.5% (*n* = 370) were alive; cardiac cause was responsible for 43.9% (*n* = 108) of the mortality in cases with known causes of death; these numbers were 40% (*n* = 524) and 34.1% (*n* = 71) in patients receiving mechanical valves, respectively.

The mean survival for the biological valve group was 102 ± 5 months, while it was 139 ± 4 months for patients with a mechanical valve, and the 10-year survival was 33% and 55% for the two groups, respectively, (*p* < 0.0001).

**Figure 2:**
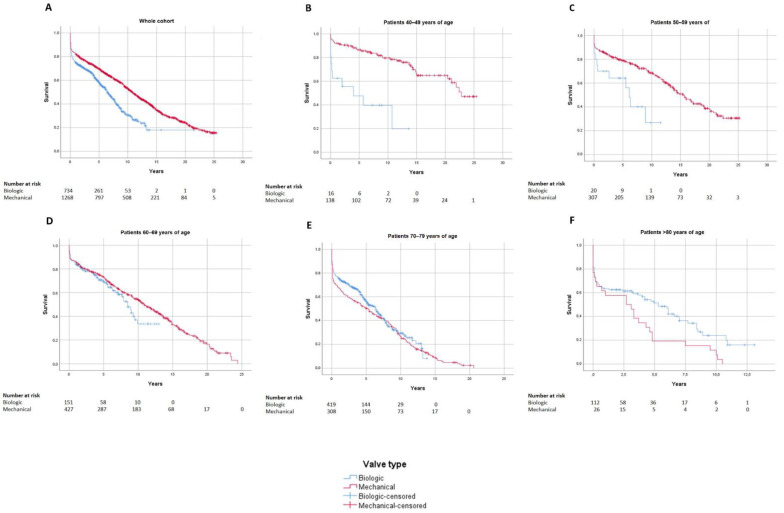
(**A**) Cumulative survival after mitral valve replacement with a biological or mechanical prosthesis in patients between 40–49 years (*p* < 0.001) (**B**), in patients between 50–59 years (*p* = 0.001) (**C**) and in patients between 60–69 years (**D**). No difference can be observed in patients between 70–79 years (**E**). No difference can be observed in patients above 80 years (*p* = 0.014) (**F**). MVR = mitral valve replacement.

Subgroup analysis based on age categories was carried out in terms of survival. A higher survival was revealed for patients with mechanical prosthesis up to the age of 60 years (<39 years: *p* = 0.047; 40–49 years: *p* < 0.0001; 50–59 years: *p* = 0.001). In the category of 60–69 years, no difference could be observed until the eighth year after surgery, while after that the mechanical group had a longer survival (*p* = 0.014). The same survival was found in the group of 70–79 years, while above 80 years, biological valves showed a higher survival (*p* = 0.014). Survival curves are shown in Figure 2.

**Figure 3:**
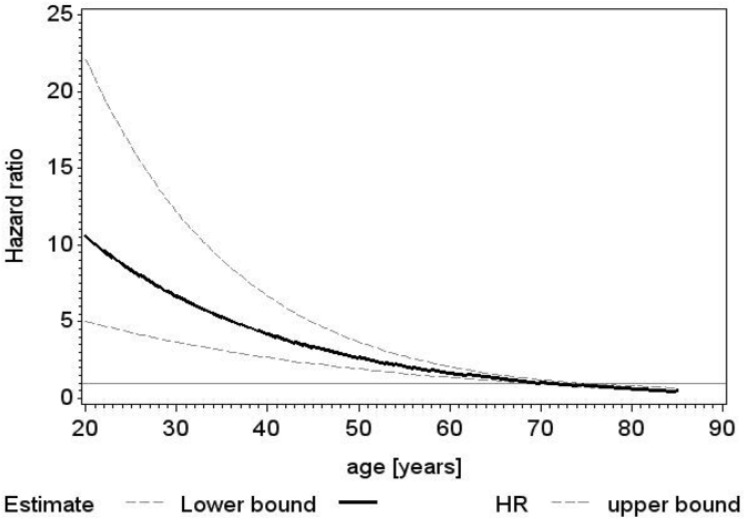
Recipients of biological prostheses compared with recipients of mechanical valves in terms of death, plotted against age as a continuous variable (black line).

Figure 3 shows the hazard ratio of death of biological valves compared to mechanical ones.

### 3.2. Postoperative Characteristics

The postoperative characteristics were assessed for 651 patients (31.7%); 319 (49%) of them were from the bioprosthesis group, while 332 (51%) were patients receiving a mechanical valve. The intraoperative characteristics are shown in Table 2. Their further cardiac and non-cardiac interventions, postoperative comorbidities, NYHA stratification and taken antithrombotic drugs are shown in Table 3.

## 4. Discussion

We present the results of 2056 mitral valve replacement surgeries carried out at Heidelberg University, Germany between 1993 and 2017.

### 4.1. Survival

Patients receiving a mechanical valve demonstrated a longer survival compared to the implantation of a biological prosthesis when evaluating the whole cohort. Subgroup analysis revealed important survival differences between the two prosthesis types in the various age groups. Mechanical valve implantation was found to result in a longer survival up to the age of 60 years, while above 80 years of age, biological prosthesis proved to have a longer survival compared to the mechanical one. Between 60 and 69 years, mechanical valves led to a longer survival from the eighth year after the operation, while no difference was observed in the age group of 70–79 years.

The results of survival in the case of biological and mechanical valves have been conflicting throughout the literature. Similar to our findings, in their multi-centre study, Goldstone et al. revealed no difference in mortality in patients aged 70–79 years when comparing biological and mechanical prostheses, while mortality was higher at the age groups of 40–49 years and 50–69 years in the case of biological valve implantation, which is also consistent with our results [8]. Our results come from a single-centre study, which means a more homogenous patient population and more uniform operative and perioperative practice. In a cohort of patients aged 18–50 years, mechanical valves led to a better survival compared to biological ones, but, interestingly, the survival benefit could not be seen in patients at the age of 18–40 years [9]. In contrast to our findings, biological valves were found to lead to a better survival in patients above 70 years, while in our cohort this benefit could be only observed above the age of 80 years. A further difference is that the authors found no survival benefit of mechanical valves under the age of 69 years [10]. On the contrary, in the study by Jamieson et al., freedom from valve-related mortality favoured mechanical prosthesis in all groups, except for patients above 70 years [11].

Further studies have been published comparing mechanical and biological valves in the controversial age groups. No survival difference could be identified in smaller studies of patients between 50–65 and under 60 years [12,13]. Chikwe and colleagues carried out a retrospective study involving 3433 patients between the age of 50 and 69 years who underwent mitral valve replacement. They did not observe any survival difference between mechanical and biological prostheses in this population [6]. These results are inconsistent with the ones in our study. Based on these published outcomes, some tendencies can be seen about the influence of the relationship between valve type and age on survival, but it is clearly shown that other factors apart from age also play a role in the outcomes after mitral valve replacement.

### 4.2. Trends in the Application of Mechanical and Biological Valves

Accordingly, in the current guidelines on valvular heart diseases, age is considered in the decision-making process between mechanical and biological prostheses as a class II recommendation. The most recent European guidelines recommend the use of mechanical valves under the age of 65 years, while the bioprosthetic valve is indicated above 70 years, and both valves are accepted between 65–70 years of age after careful consideration of other relevant factors [5]. The recommendations by the American guidelines favour a mechanical valve under the age of 65 years, and a biological one above 65 years [14].

The use of a biological valve was relatively low in our patient cohort before the year 2003, and after that it started to show an increasing tendency, leading to being more frequently implanted than mechanical valves. These changes are consistent with the ones found in the literature. In the United States, the use of biological valves for mitral valve replacement doubled between 1998 and 2005, and since 2003 more tissue valves are used than mechanical ones in the mitral position in patients 75 years or older. Their use also tripled in patients under 65 years [15]. In the paper from Schnittmann and colleagues, bioprosthetic mitral replacements in patients 18–50 years of age showed an increase from 10% to nearly 34% between 1997 and 2014, and up to one-third of these patients received bioprosthetic valves [9]. The reasons behind this trend can be the increasing durability of bioprosthetic valves [16] and also the published articles reporting similar survival between valve types [17,18]. When analysing the age groups in our cohort, patients between the ages of 50–59 years showed only a slight increase, while the group of 60–69-year-old patients demonstrated remarkable growth in the number and proportion of implanted biological valves. These trends are justified by the above discussed guidelines, but, importantly, in the age group of 60–69 years, our results demonstrated a higher survival after the eighth year of operation in the case of mechanical valves.

### 4.3. Postoperative Outcomes

Besides survival, another aspect that can aid decision making in prosthesis-type selection is the difference in postoperative outcomes. The most often evaluated complications after mitral valve replacement are major bleeding, stroke and the need for reoperation.

A major drawback of a biological valve compared to a mechanical one is its association with more frequent reoperation [6,8,9,11]. Bioprostheses are prone to structural valve degeneration, which limits their durability and, therefore, leads to reoperation generally 10–15 years after the surgery [19]. In our cohort, no difference was observed in reoperation between the two prosthesis types. However, we identified a significantly higher rate of postoperative bleeding in the case of mechanical valve implantation, which is mostly in agreement with other studies [6,8]. Furthermore, there was a tendency towards more frequent stroke occurrence in patients with implanted mechanical valves in our cohort.

Postoperatively, a significantly higher number of patients in our study were stratified in the NYHA class I functional category in the cohort of mechanical valves, while significantly more patients with bioprosthesis belonged to NYHA class II and NYHA class III categories, which could be an indicator for better functional outcomes in the case of mechanical valve implantation.

As expected, significantly more patients with an implanted mechanical valve took vitamin K antagonists, since mechanical valves require life-long anticoagulation therapy with this type of medication to avoid the occurrence of thrombosis and thromboembolic events [20]. The use of non-vitamin K antagonist oral anticoagulant (NOAC) in the mechanical prosthesis group was only 0.3%, as these drugs are contraindicated in this group of patients [5]. A major drawback of mechanical valves compared to biological ones is the need for life-long anticoagulation. However, as our cohort shows, a high number of patients with biological valves also take anticoagulants with certain indications. The use of NOAC is significantly more common in our bioprosthesis group due to the more frequent atrial fibrillation. The fact that anticoagulants are also frequently indicated for these patients makes the question of anticoagulation less relevant, thereby diminishing an important advantage of biological prosthesis in our cohort. A further difference between the two valve groups in terms of antithrombotic therapy was the significantly more common use of antiplatelet medications in the biological valve cohort, which could be explained by the higher number of peripheral artery disease in these patients, in which condition, antiplatelet medication is indicated [21].

### 4.4. Limitations

Our results come from a single-centre study, which means a more homogenous patient population and more uniform operative and perioperative practice. It must be emphasised that our mechanical and biological valve cohorts had significant preoperative differences that are likely to have contributed to our results. Importantly, according to the current recommendations, patients receiving bioprosthesis were older and, consequently, they presented with more comorbidities, which included systemic and pulmonary hypertension, hyperlipoproteinemia, diabetes mellitus, peripheral arterial disease, carotid stenosis and history of stroke. On the other hand, the mechanical valve group had significantly more patients with liver enlargement and peripheral oedema, which could demonstrate the presence of a more severe mitral valve disease.

Further limitations of our results are that this study has a retrospective single-centre design and that the cause of death is unknown for a relative high number of patients in both the biological and mechanical valve groups.

## 5. Conclusions

Despite the fact that mitral valve repair surgery has made great advances in recent years, mitral valve replacement still plays a pivotal role in the management of mitral valve disease. Making decisions between biological and mechanical prostheses is challenging because the long-term survival and other complications are not well-defined and results are conflicting. Previous retrospective studies suggested that biological prostheses might be a reasonable alternative to mechanical prosthetic valve replacement in younger patients (aged 50 to 70 years). In our cohort, mechanical valves led to a higher survival up to the age of 60, while biological ones had a longer survival only in patients above 80 years.

## Figures and Tables

**Table 1:** Preoperative characteristics.

	Total Cohort	Biological	Mechanical	*p* Value
Pulmonary stasis	299/2034 (14.7%)	123/743 (16.6%)	176/1291 (13.6%)	0.073
Liver enlargement	205/2021 (10.1%)	49/738 (6.6%)	156/1283 (12.2%)	*<0.001*
Peripheral oedema	519/2037 (25.5%)	156/744 (21.0%)	363/1293 (28.1%)	*<0.001*
Embolic accidents	135/2032 (6.6%)	31/747 (4.1%)	104/1285 (8.1%)	*<0.001*
Cardiac decompensation	691/2038 (33.9%)	267/747 (35.7%)	424/1291 (32.8%)	0.183
Stroke	219/1578 (13.9%)	107/727 (14.7%)	112/851 (13.2%)	0.373
Diabetes mellitus	484/2033 (23.8%)	207/747 (27.7%)	277/1286 (21.5%)	*0.002*
Hyperlipoproteinemia	864/1992 (43.4%)	374/741 (50.5%)	490/1251 (39.2%)	*<0.001*
Hypertension	1390/2017 (68.9%)	606/745 (81.3%)	784/1272 (61.6%)	*<0.001*
Pulmonary hypertension	837/1575 (53.1%).7	401/726 (55.2%)	436/849 (51.4%)	0.124
Peripheral arterial disease	116/1579 (7.3%)	58/726 (8.0%)	58/853 (6.8%)	0.367
Carotid stenosis	128/1573 (8.1%)	65/725 (9.0%)	63/848 (7.4%)	0.267
History of smoking	554/1809 (30.6%)	190/710 (26.8%)	364/1099 (33.1%)	*0.004*
**Etiology**				
Endocarditis	287/2056 (14.0%)	126/748 (16.8%)	161/1308 (12.3%)	*0.004*
Stenosis	208/2056 (10.1%)	46/748 (6.1%)	162/1308 (12.4%)	*<0.001*
Insufficiency	1390/2056 (67.6%)	567/748 (75.8%)	823/1308 (62.9%)	*<0.001*
Stenosis+ Insufficiency	458/2056 (22.3%)	135/748 (18.0%)	323/1308 (24.7%)	*<0.001*
**Antithrombotic Therapy**				
Aspirin	466/2012 (23.2%)	248/740 (33.5%)	218/1272 (17.1%)	*<0.001*
Other platelet aggregation inhibitors	33/1798 (1.8%)	28/626 (4.5%)	5/1172 (0.4%)	*<0.001*
Anticoagulants	762/2014 (37.8%)	237/740 (32.0%)	525/1274 (41.2%)	*<0.001*
**NYHA Classification**				
NYHAI	53/2053 (2.6%)	9/746 (1.2%)	44/1307 (3.4%)	*0.003*
NYHAII	301/2053 (14.7%)	113/746 (15.1%)	188/1307 (14.4%)	0.638
NYHAIII	1142/2053 (55.6%)	453/746 (60.7%)	689/1307 (52.7%)	*<0.001*
NYHAIV	557/2053 (27.1%)	171/746 (22.9%)	386/1307 (29.5%)	*<0.001*

The patients’ characteristics before the mitral valve replacement are shown.

**Table 2:** Intraoperative characteristics.

	Total Cohort	Biological	Mechanical	*p* Value
**Approach**				
Median sternotomy	1937/2049 (94.5%)	690/748 (92.2%)	1247/1301 (95.8%)	*<0.001*
MIC	112/2049 (5.5%)	58/748 (7.8%)	54/1301 (4.2%)	*<0.001*
**Valve characteristics**				
Size	31 (29–31)	29 (29–31)	31 (29–32)	<0.001 *
Types (*n*)		Carpentier-Edwards Physio II 5200 (2)	St. Jude Medical Standard 101 (934)	
		Edwards Lifesciences Perimount + 6900 TFX (2)	St. Jude Medical Masters 501/505 (303)	
		Epic Supra ESP E100 (5)	St. Jude Medical HP-Serie 105 (4)	
		Hancock II T505 (39)	Medtronic Hall A/M 7700 (58)	
		Hancock II T510 (672)	Duran 601H/608H (1)	
		Perimount Magna Mitral Ease 7300 TFX (18)	Carpentier-Edwards Classic 4400/4500 (3)	
		Sorin Pericarbon More (2)	Other bileaflet valves (4)	
		St. Jude Medical Epic ELS (6)		

The intraoperative characteristics are presented. * Mann–Whitney U Test.

**Table 3:** Postoperative characteristics.

	Total Cohort	Biological	Mechanical	*p* Value
Cardiac death *	179/454 (39.4%)	108/246 (43.9%)	71/208 (34.1%)	*0.034*
Myocardial infarction	36/651 (5.5%)	17/319 (5.3%)	19/332 (5.7%)	0.826
Reoperation	10/651 (1.5%)	5/319 (1.6%)	5/332 (1.5%)	0.949
Other valve replacement	13/651 (2%)	4/319 (1.3%)	9/332 (2.7%)	0.184
Bypass surgery	4/651 (0.6%)	2/319 (0.6%)	2/332 (0.6%)	0.968
Pacemaker or Defibrillator	134/651 (20.6%)	67/319 (21%)	67/332 (20.2%)	0.970
PTCA ^1^ or stents	19/651 (2.9%)	9/319 (2.8%)	10/332 (3.0%)	0.885
Aortic operation	1/651 (0.2%)	1/319 (0.3%)	0	0.49 **
Ablation	19/651 (2.9%)	7/319 (2.2%)	12/332 (3.6%)	0.282
Other cardiac intervention	15/651 (2.3%)	9/319 (2.8%)	6/332 (1.8%)	0.743
Kidney disease	167/651 (25.7%)	100/319 (31.3%)	67/332 (20.2%)	*0.001*
Embolic or thrombotic incidence	48/651 (7.4%)	29/319 (9.1%)	19/332 (5.7%)	0.100
Postoperative bleeding	53/651 (8.1%)	15/319 (4.7%)	38/332 (11.4%)	*0.002*
Stroke	93/650 (14.3%)	38/318 (11.9%)	55/332 (16.6%)	0.090
AV block postop	28/651 (4.3%)	13/319 (4.1%)	15/332 (4.5%)	0.781
Atrial fibrillation	384/651 (59.0%)	214/319 (67.1%)	170/332 (51.2%)	*<0.001*
**NYHA**				
NYHAI	298/651 (45.8%)	110/319 (34.5%)	188/332 (56.6%)	*<0.001*
NYHAII	193/651 (29.6%)	117/319 (36.7%)	76/332 (22.9%)	*<0.001*
NYHAIII	144/651 (22.1%)	83/319 (26%)	61/332 (18.4%)	*0.019*
NYHAIV	16/651 (2.5%)	9/319 (2.8%)	7/332 (2.1%)	0.557
**Antithrombotic therapy**				
Vitamin K antagonist	484/645 (75.0%)	160/319 (50.2%)	324/326 (99.4%)	*<0.001*
NOAC ^2^	64/651 (9.8%)	63/319 (19.7%)	1/332 (0.3%)	*<0.001*
LMWH ^3^	14/651 (2.2%)	8/319 (2.5%)	6/332 (1.8%)	0.538
Platelet aggregation inhibitors	74/651 (11.4%)	72/319 (22.6%)	2/332 (0.6%)	*<0.001*

Postoperative characteristics of the patients are shown. * Compared to the number of deaths with a known cause. ** Calculated with Fisher exact test. ^1^ Percutaneous transluminal coronary angioplasty; ^2^ novel oral anticoagulants; ^3^ low molecular weight heparin.

## Data Availability

The data underlying this article will be shared on reasonable request to the corresponding author.

## Abbreviations

NOAC: Non-vitamin K antagonist oral anticoagulant

NYHA: New York Heart Association
